# Supplementary figures and images for: Cytokines reprogram airway sensory neurons in asthma
Source: bioRxiv. 2024 Sep 18:2023.01.26.525731. Preprint. [Version 2] doi: 10.1101/2023.01.26.525731 (PMC11429693; doi:10.1101/2023.01.26.525731)

**A**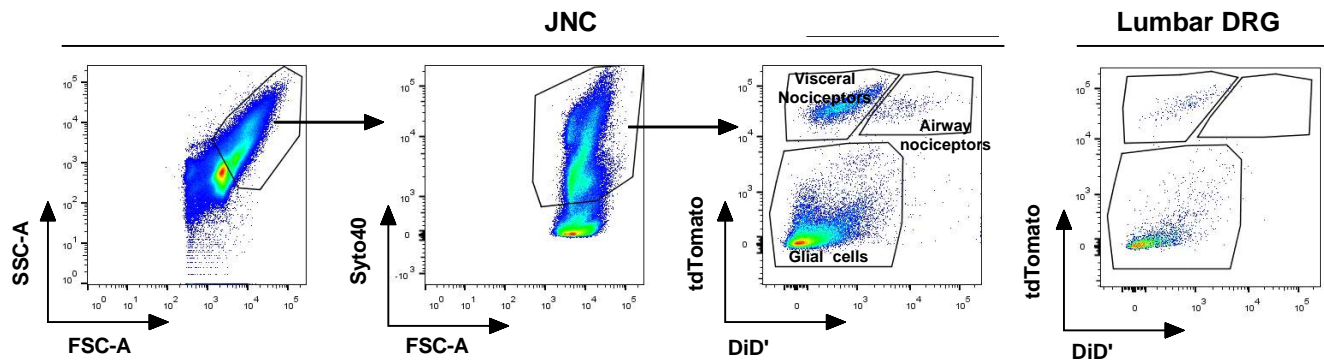**B**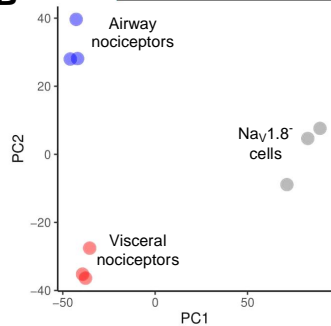**C**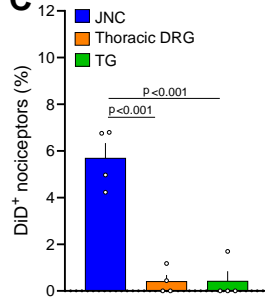**D**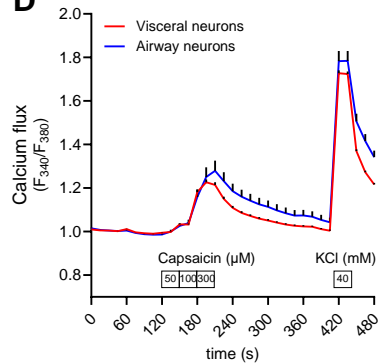**E**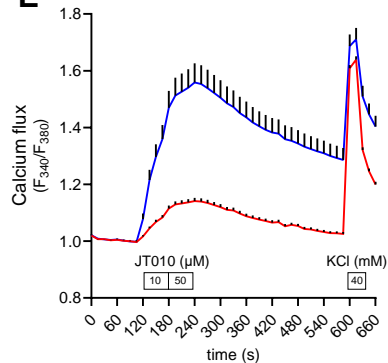

Supplement: Supplement 1 [file media-1.pdf]

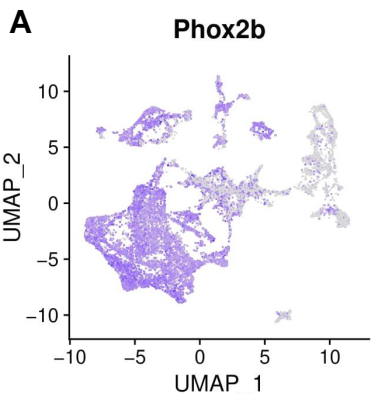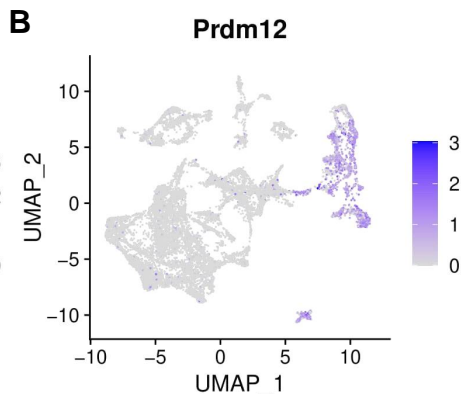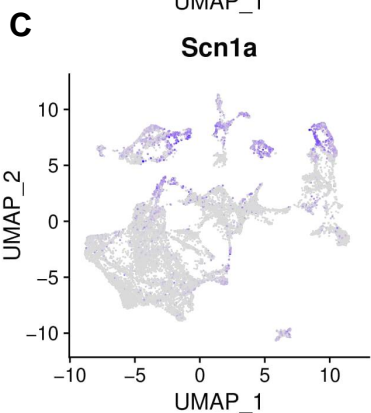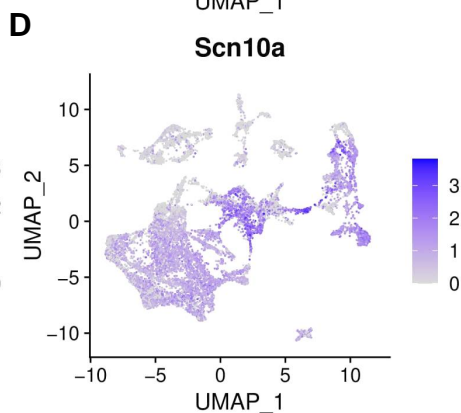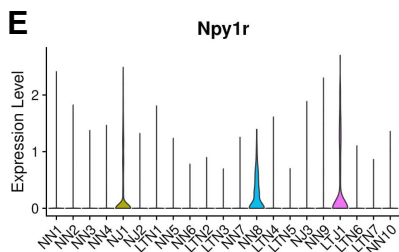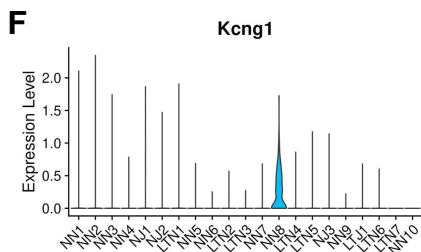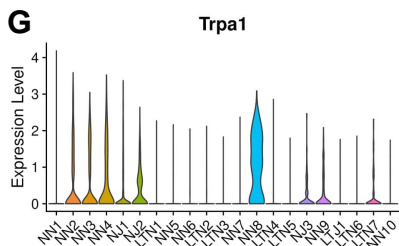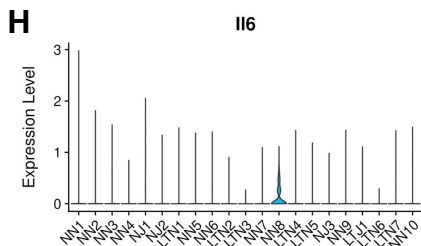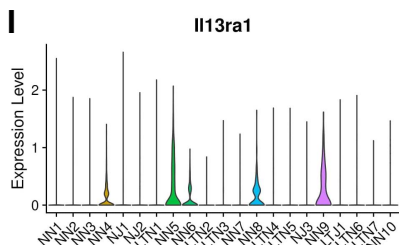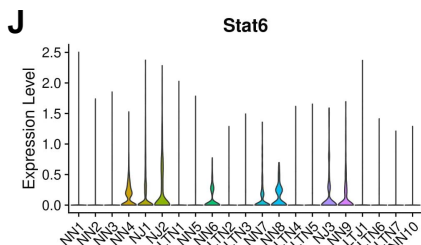

Supplement: Supplement 2 [file media-2.pdf]

**A**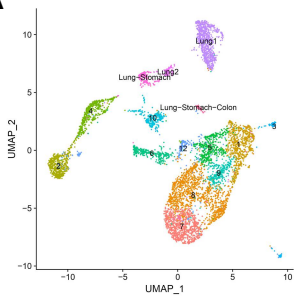**B**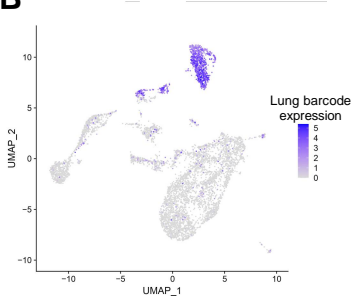**C****Npy1r**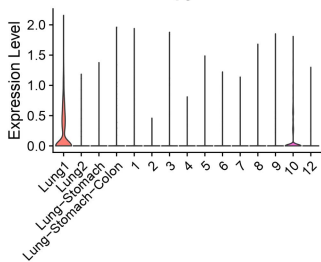**D****Kcng1**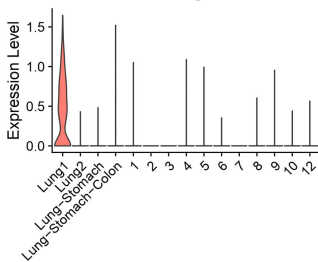**E****Trpa1**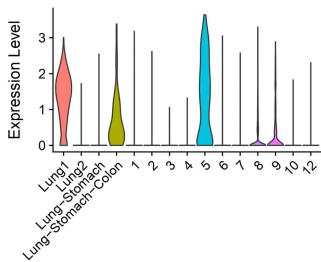**F****Il6**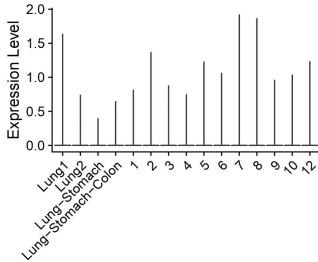**G****Il13ra1**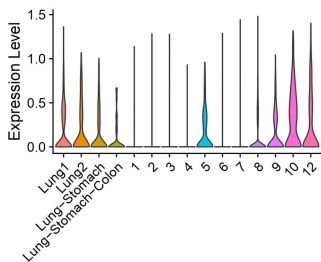**H****Scn10a**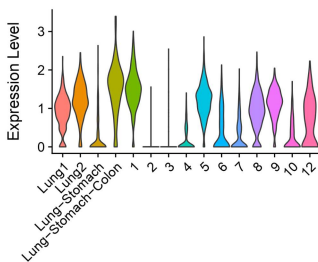

Supplement: Supplement 3 [file media-3.pdf]

**A**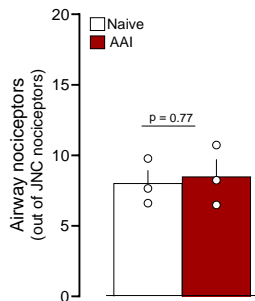**B**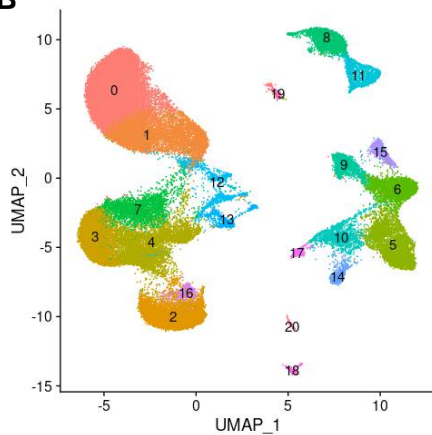**C**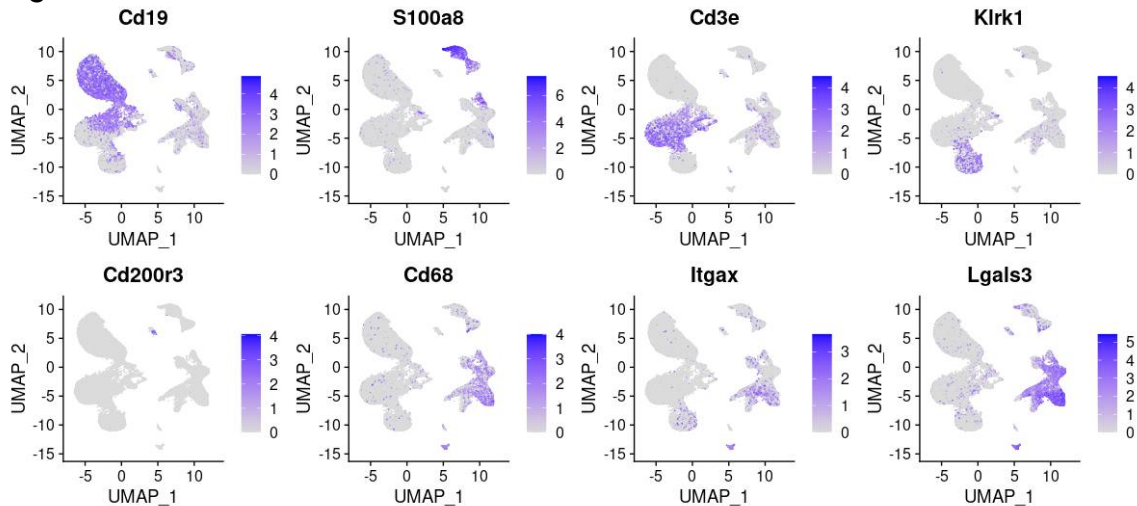

Supplement: Supplement 4 [file media-4.pdf]

**A**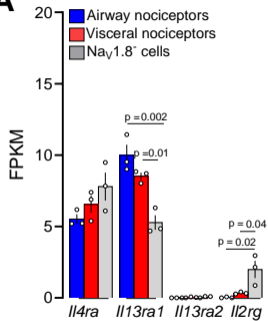**B**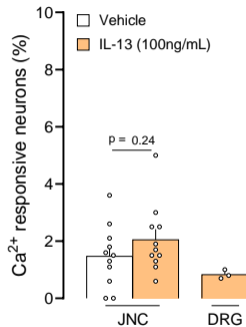**C**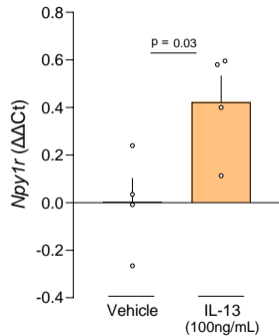

Supplement: Supplement 5 [file media-5.pdf]

**A**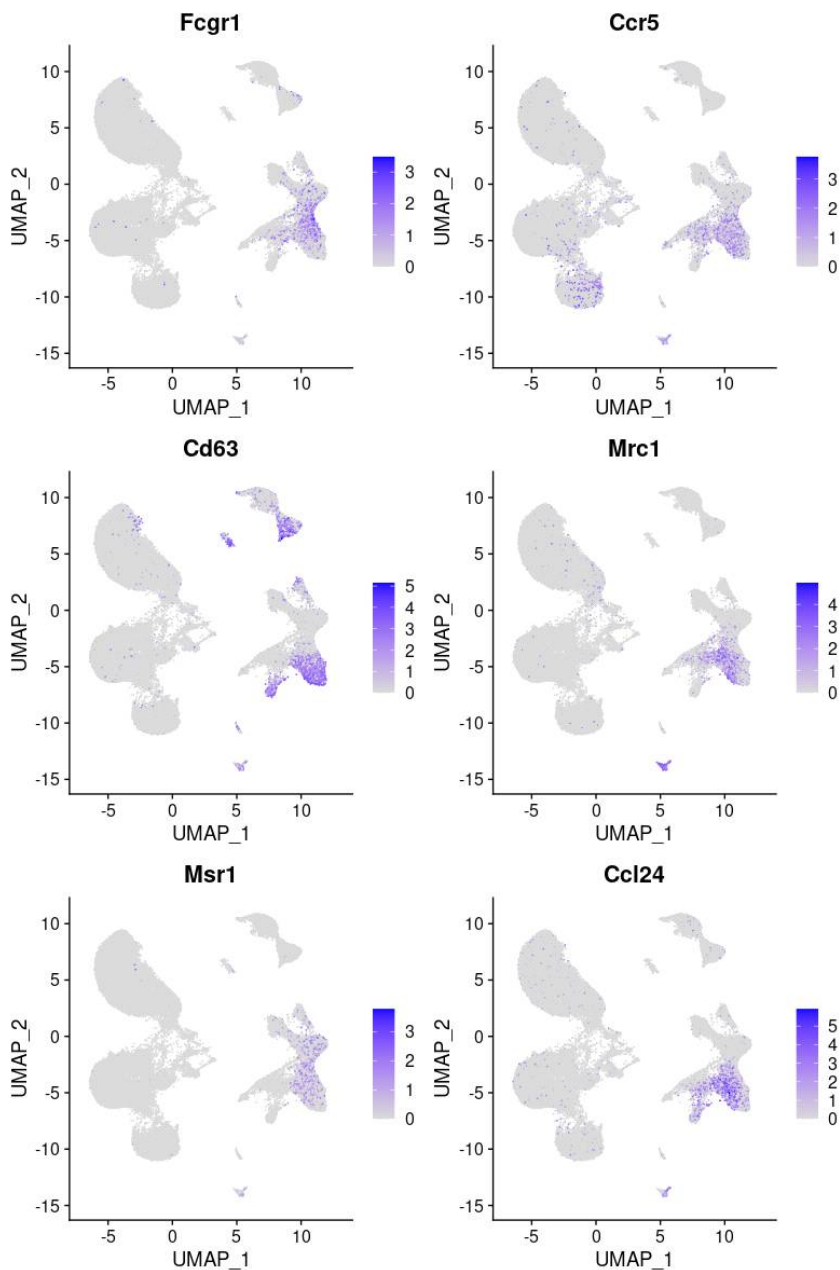

Supplement: Supplement 6 [file media-6.pdf]

**A**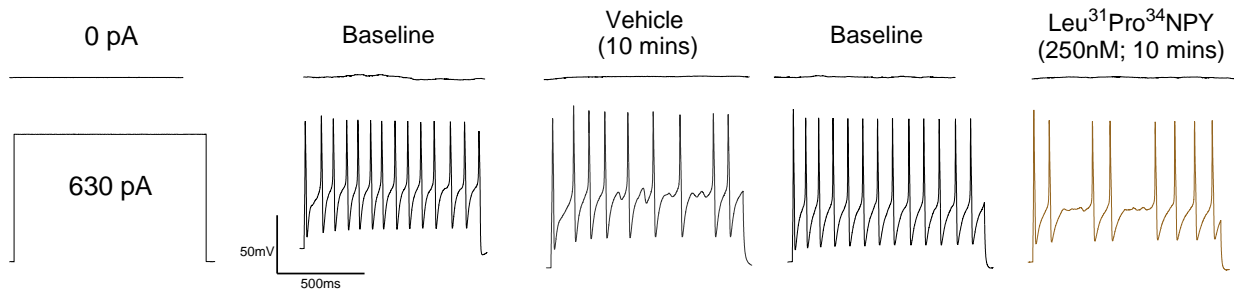**B**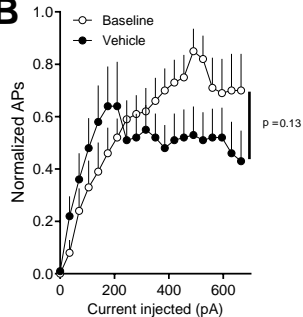**C**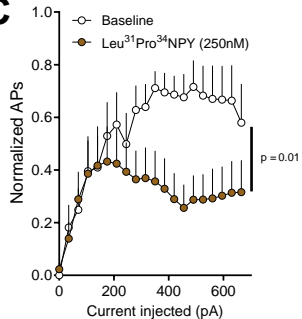

Supplement: Supplement 7 [file media-7.pdf]

**A**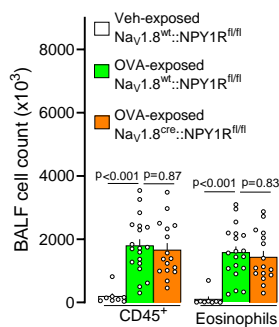**B**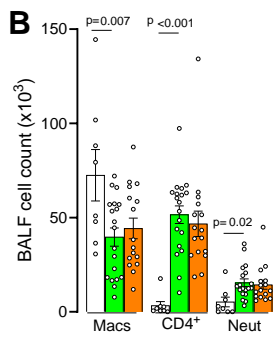**C**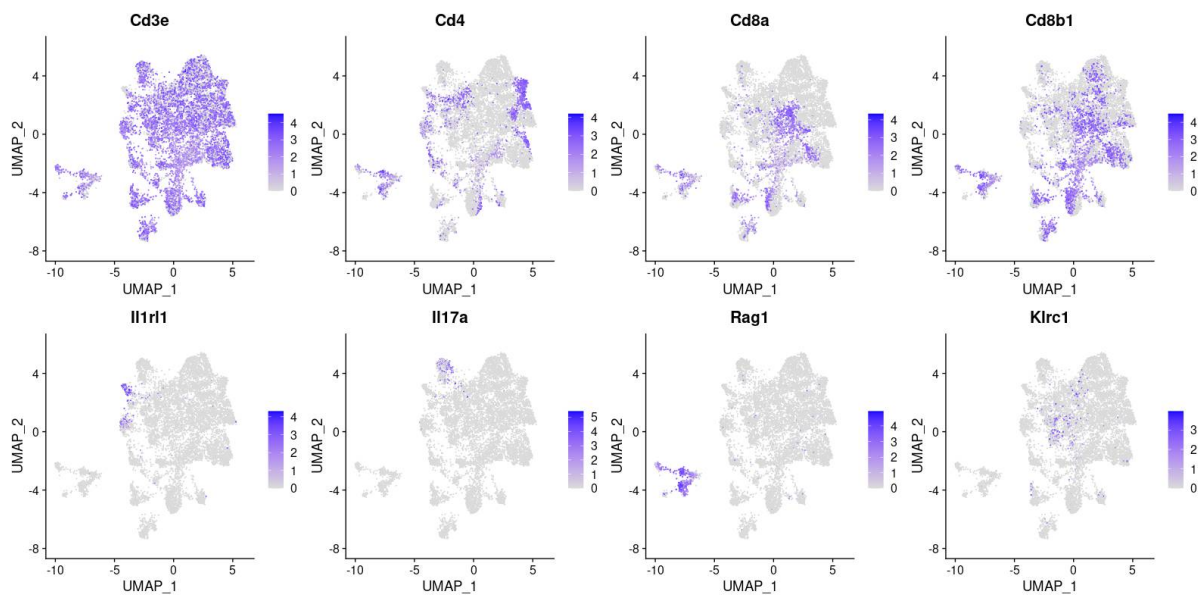**D**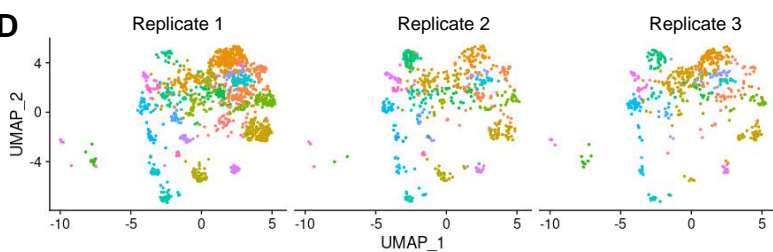**E**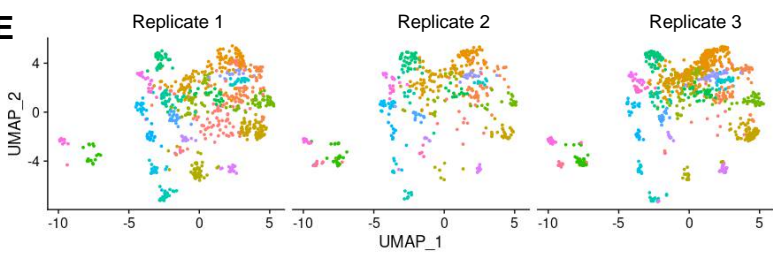**F**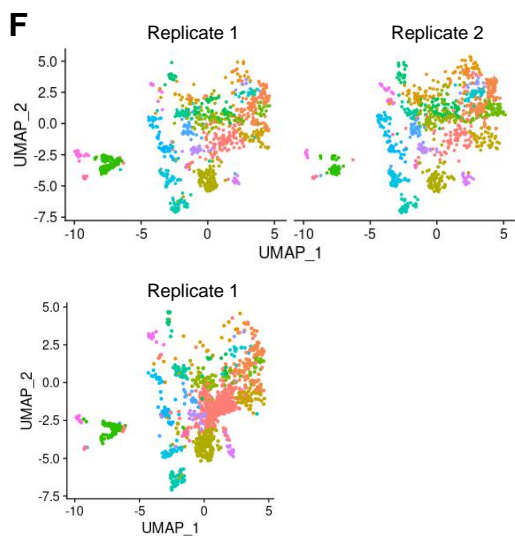**G**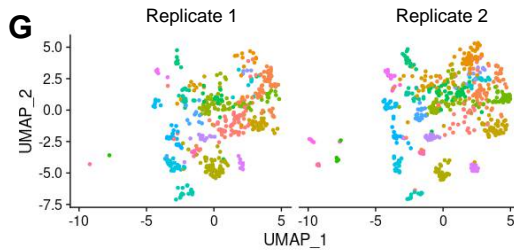

Supplement: Supplement 8 [file media-8.pdf]
