# Supplementary material for: Cytokines reprogram airway sensory neurons in asthma
Source: bioRxiv. 2024 Sep 18:2023.01.26.525731. Preprint. [Version 2] doi: 10.1101/2023.01.26.525731 (PMC11429693; doi:10.1101/2023.01.26.525731)
Supplement: Supplement 19 [file NIHPP2023.01.26.525731v2-supplement-19.pdf]

bioRxiv preprint doi: https://doi.org/10.1101/2023.01.26.525731; this version posted September 18, 2024. The copyright holder for this preprint (which was not certified by peer review) is the author/funder, who has granted bioRxiv a license to display the preprint in perpetuity. It is made available under aCC-BY 4.0 International license.

Supplementary Figure 1. Airway vagal nociceptor neurons have a unique transcriptome.

(A) Jugular nodose complex neurons gating strategy. Small debris were eliminated (FSC/SSC), and the whole cells were identified (nucleus marker SYTO40). Populations of airway-innervating nociceptor neurons ($Na_V1.8^+DiD^+$), visceral nociceptors ($Na_V1.8^+DiD^-$) and $Na_V1.8^-$ cells were then separated. Lumbar DRG were used as gating controls since they do not innervate the airways **(A)**.

(B) The transcriptome of purified airway-innervating nociceptor neurons ($Na_V1.8^+DiD^+$), visceral nociceptors ($Na_V1.8^+DiD^-$) and glial cells ($Na_V1.8^-DiD^-$) was analyzed by RNA sequencing and population segregation was confirmed using principal component analysis **(B)**.

(**C**) Naive 8-week-old male and female $Na_V1.8^{cre}$::$tdTomato^{fl/wt}$ mice were injected intranasally with the retrograde tracer DiD' (200 μM). Fourteen days later, the mice were euthanized and JNC, thoracic DRG, and TG ganglia isolated, dissociated, and imaged with a fluorescence microscope. DiD' retrotracer was detected in JNC nociceptor neurons but virtually absent in other ganglia (**C**).

(**D-E**) Naive 8-week-old male and female C57BL6 mice were injected intranasally with the retrograde tracer DiD' (200 μM). Fourteen days later, the mice were euthanized and their JNC ganglia isolated and dissociated. JNC neurons were cultured (16 h) and calcium responsiveness to noxious stimuli was assessed. While the average neuronal responsiveness to the TRPV1 agonist capsaicin (300 nM) was stable between the two groups (**D**), the calcium flux induced by the TRPA1 agonist JT010 (50 μM) was higher in airway-innervating nociceptor neurons (**E**).

*Data are shown as flow cytometry dot plot (**A**), principal component analysis (**B**), and mean ± S.E.M (**C–E**). N are as follows: **C**: n=4 culture dishes**,** **D**: n=116 airway-innervating neurons and 1307 visceral neurons, **E**: n=137 airway-innervating neurons and 1406 visceral neurons. P-values were determined by a two-sided unpaired Student's t-test (**C**) and are indicated in the figure.*

bioRxiv preprint doi: https://doi.org/10.1101/2023.01.26.525731; this version posted September 18, 2024. The copyright holder for this preprint (which was not certified by peer review) is the author/funder, who has granted bioRxiv a license to display the preprint in perpetuity. It is made available under aCC-BY 4.0 International license.

Supplementary Figure 2. JNC airway nociceptor neuron markers

(**A-D**) UMAPs of *Slc17a6*$^{+}$ (VGLUT2) JNC neurons from single-cell RNA sequencing showing expression of *Phox2b* (**A**), *Prdm12* (**B**), *Scn1a* (**C**), and *Scn10a* (**D**). *Phox2b* and *Prdm12* delineate the nodose and jugular neurons, while *Scn1a* and *Scn10a* delineate low-threshold sensory neuron and nociceptor neuron populations. The experimental details were defined in *Prescott et al.* and the bioinformatic analysis is described in the methods section (**E**).

(**E–J**) Violin plot showing expression of *Npy1r* (**E**), *Kcng1* (**F**), *Trpa1* (**G**), *Il6* (**H**), *Il13ra1* (**I**), *Stat6* (**J**) in neuronal cells for each cluster identified by single-cell sequencing. All these genes are co-expressed in the airway-specific nociceptor neuron cluster NN8. The experimental details were defined in *Prescott et al.* and the bioinformatic analysis is described in the methods section.

*Data are shown as UMAPs with log-normalized expression as a feature (**A-D**) or as a violin plot of the log-normalized expression (**E-J**).*

bioRxiv preprint doi: https://doi.org/10.1101/2023.01.26.525731; this version posted September 18, 2024. The copyright holder for this preprint (which was not certified by peer review) is the author/funder, who has granted bioRxiv a license to display the preprint in perpetuity. It is made available under aCC-BY 4.0 International license.

Supplementary Figure 3. Lung projecting neurons single cell RNA sequencing.

(**A-B**) UMAPs of barcode positive JNC neurons cluster from single-cell RNA projection-sequencing[7] (**A**) and lung barcode expression (**B**). 4 clusters innervate the lung, 2 of them being lung specific, the two others also innervating stomach and colon (**B**). The experimental details were defined in Zhao *et al*[7]. and the bioinformatic analysis is described in the methods section.

(**C-H**) Violin plot showing expression of *Npy1r* (**C**), *Kcng1* (**D**), *Trpa1* (**E**), *Il6* (**F**), *Il13ra1* (**G**), *Scn10a* (**H**) in neuronal cells for each cluster identified by single-cell sequencing.

*Data are shown as UMAPs with log-normalized expression as a feature (**A-B**) or as a violin plot of the log-normalized expression (**C-H**).*

bioRxiv preprint doi: https://doi.org/10.1101/2023.01.26.525731; this version posted September 18, 2024. The copyright holder for this preprint (which was not certified by peer review) is the author/funder, who has granted bioRxiv a license to display the preprint in perpetuity. It is made available under aCC-BY 4.0 International license.

Supplementary figure 4. Single cell RNA seq reveals immune cells heterogeneity upon AAI.

(**A**) 8-week-old male and female nociceptor neuron reporter (Na$_V$1.8^{cre}::tdTomato$^{fl/wt}$) mice underwent the ovalbumin mouse models of asthma. Allergic inflammation was induced in mice by an initial sensitization to ovalbumin (OVA) (i.p. day 0 and 7) followed by inhaled OVA challenges (days 14–17). On days 2, 3, and 4, mice were injected intranasally with the retrograde tracer DiD' (200 µM). One day after the last allergen challenge, the mice were euthanized and their JNCs isolated and dissociated, and airway-innervating nociceptor neurons (Na$_V$1.8$^+$DiD$^+$) were purified by flow cytometry (**A**).The number of JNC airway-innervating nociceptor neurons is similar between naïve and OVA-exposed mice (tdTomato$^+$DiD$^+$) (**A**).

(B-C) 8-week-old male and female C57BL6 mice underwent the ovalbumin model of asthma. Lungs were harvested, immune cells sorted by flow cytometry before analysis by scRNA-seq. 21 different subtypes of immune cells were found in the airways **(E)**. Various specific markers allow to identify those cell types including B cells (*Cd19*+), T cells (*Cd3e*+), Granulocytes (*S100ab*+), NK cells (*Klrk1*+), Basophils (*Cd200r3*+), and Antigen Presenting Cells (APC, which include Macrophages and Dendritic cells, with varying expression of *Cd68*, *Itgax* and *Lgals3*). **(F)**.

*Data are shown as mean ± S.E.M (**A**), Umap **(B),** or Umap displaying Seurat normalized gene expression for the indicated genes (**C**)**.** N are as follows: **A**: n=3 biological replicates (4 mice per sample), **B-C**: n=1-3 biological replicates (2 mice per sample). P-values were determined by a two-sided unpaired Student's t-test (**A**).*

bioRxiv preprint doi: https://doi.org/10.1101/2023.01.26.525731; this version posted September 18, 2024. The copyright holder for this preprint (which was not certified by peer review) is the author/funder, who has granted bioRxiv a license to display the preprint in perpetuity. It is made available under aCC-BY 4.0 International license.

Supplementary figure 5. IL-13 reprogram airway nociceptor neurons through IL4RII.

(**A**) Naive 8-week-old male and female nociceptor neurons reporter (NaV1.8^{cre}::tdTomato$^{fl/wt}$) mice were injected intranasally with the retrograde tracer DID' (200 μM). Fourteen days later, the mice were euthanized and their JNC ganglia isolated and dissociated. Airway-innervating nociceptor neurons (NaV1.8^{+}DiD^{+}), visceral nociceptors (NaV1.8^{+}DiD^{-}) and NaV1.8^{-} cells were purified by flow cytometry and RNA sequenced. Both IL4RII subunits, *Il4ra* and *Il13ra1*, were detected in JNC nociceptors. Other IL-13 and IL-4 receptors are not detected. *IL13ra1* transcript expression was higher in nociceptor neurons when compared to levels measured in NaV1.8^{-} cells (**A**).

(B) Naive 8-week-old male and female C57Bl6 mice were euthanized and their JNC and DRG ganglia were isolated and cultured (16 h). The neurons were then loaded with the calcium indicator Fura-2AM (5 μM) and their responsiveness to IL-13 (100 ng/mL) was assessed using calcium microscopy. DRG and JNC neurons show limited calcium response when exposed to IL-13 compared to its vehicle **(B)**.

(**C**) Naive 8-week-old male and female C57BL6 mice DRG neurons were isolated and cultured in the presence of IL-13 (100 ng/mL; 24 h) or its vehicle. Transcript levels were assessed by qPCR. In comparison to the vehicle, IL-13 increased DRG neurons' expression of *Npy1r* (**C**).

*Data are shown as mean ± S.E.M (**A-B**), or as box (25th-75th percentile) and whisker (min-to-max) plots (**C**). N are as follows: **A**: n=3 biological replicates (4 mice per sample), **B**: n=11 culture dishes for JNC and 3 culture dishes for DRG, **C**: n=4 cultures from different mice per group. P-values were determined by one-way ANOVA with post hoc Tukey's (**A**); or a two-sided unpaired Student's t-test (**B-C**). P-values are shown in the figure.*

bioRxiv preprint doi: https://doi.org/10.1101/2023.01.26.525731; this version posted September 18, 2024. The copyright holder for this preprint (which was not certified by peer review) is the author/funder, who has granted bioRxiv a license to display the preprint in perpetuity. It is made available under aCC-BY 4.0 International license.

Supplementary Figure 6. NPY is expressed in M2 macrophages.

(**A-B**) Eight-week-old female mice were subjected to the ovalbumin-induced asthma model. Mice were initially sensitized to ovalbumin (OVA) intraperitoneally (i.p.) on days 0 and 7, followed by inhaled OVA challenges from days 14 to 17. On day 18, lungs were harvested and $CD45^+$ cells were sorted by flow cytometry. Their transcriptomes were analyzed by single-cell RNA sequencing. Related to Figure 6, showing that NPY is expressed in the cluster of myeloid cells including Macrophages and Denditic cells, we show here that the NPY positive population also express M2 macrophages markers (*Fcgr1, Ccr5, Cd63, Mrc1, Msr1* and *Ccl24*).

bioRxiv preprint doi: https://doi.org/10.1101/2023.01.26.525731; this version posted September 18, 2024. The copyright holder for this preprint (which was not certified by peer review) is the author/funder, who has granted bioRxiv a license to display the preprint in perpetuity. It is made available under aCC-BY 4.0 International license.

Supplementary Figure 7. NPY1R blunts JNC nociceptors excitability.

(A-C) 8-week-old male and female NPY1R reporter (NPY1R^{cre}::tdTomato$^{fl/wt}$) mice were sacrificed and their JNC neurons harvested and cultured (16 hours). Whole cell patch clamp electrophysiology was performed on the NPY1R^{+} nociceptor neurons. A current clamp was applied while the neurons' membrane potential was recorded before and after exposing (10 min) the cell to Leu^{31}Pro^{34}NPY (250 nM) or its vehicle. The number of action potentials for each neuron was normalized by the maximum number observed at baseline (**B-C**. While the vehicle had little to no effect on neuronal excitability (**A**, **B**), Leu^{31}Pro^{34}NPY reduced the number of action potentials in response to current stimulation in NPY1R^{+} (tdTomato) neurons **(A, C).**

*Data are shown as traces of membrane potential for individual neurons (**A**), mean ± S.E.M **(B, C)**, or box (25th–75th percentile) and whisker (min-to-max) plots **(D-E)**. N are as follows: **B, D**: n=10 vehicle treated neurons**, C, D:** n=9 Leu^{31}Pro^{34}NPY treated neurons, **E**: n=19 culture wells. P-values were determined by two-way ANOVA (**B, C**) or two-sided unpaired Student's t-test **(D-E)**. P-values are shown in the figure.*

bioRxiv preprint doi: https://doi.org/10.1101/2023.01.26.525731; this version posted September 18, 2024. The copyright holder for this preprint (which was not certified by peer review) is the author/funder, who has granted bioRxiv a license to display the preprint in perpetuity. It is made available under aCC-BY 4.0 International license.

Supplementary Figure 8. NPY1R does not affect immune cell infiltration but changes T cells profile.

(**A-B**) Eight-week-old female nociceptor neuron NPY1R conditional knockout ($Na_V1.8^{cre}$::$NPY1R^{fl/fl}$) and littermate control mice ($Na_V1.8^{wt}$::$NPY1R^{fl/fl}$) were subjected to the ovalbumin-induced asthma model. Mice were initially sensitized to ovalbumin (OVA) intraperitoneally (i.p.) on days 0 and 7, followed by inhaled OVA challenges from days 14 to 17. One day after the last allergen challenge, bronchoalveolar lavage fluid (BALF) was collected and immunophenotyped using flow cytometry. OVA-exposed mice demonstrated significant airway inflammation with increased infiltration of leukocytes ($CD45^+$), including eosinophils ($CD45^+CD11C^{low}SiglecF^{Hi}$), neutrophils ($CD45^{pos}SiglecF^{low}CD11B^{high}LY6G^{high}$), and CD4 T cells ($CD45^{pos}SiglecF^{low}CD11B^{low}CD4^{high}$), while the number of alveolar macrophages ($CD45^+CD11C^{high}SiglecF^{Hi}$) decreased. The conditional knockout of NPY1R did not affect BALF cell numbers (**A-B**).

(**C-H**) Eight-week-old female C57BL/6 mice, either pretreated with Resiniferatoxin or untreated, together with nociceptor neuron NPY1R conditional knockout mice ($Na_V1.8^{cre}$::$NPY1R^{fl/fl}$) and their littermate controls ($Na_V1.8^{wt}$::$NPY1R^{fl/fl}$), were subjected to the ovalbumin-induced asthma model. On day 18, lungs were harvested and $CD45^+$ cells were sorted by flow cytometry. Their transcriptomes were analyzed by single-cell RNA sequencing. T-cells were subclustered, and various markers were used to classify the different subpopulations. UMAPs from each biological replicate of OVA-exposed $Na_V1.8^{wt}$::$NPY1R^{fl/fl}$ (**D**), $Na_V1.8^{cre}$::$NPY1R^{fl/fl}$ (**E**), C57BL6 mice treated with vehicle (**F**), C57BL6 mice treated with RTX (**G**), and naive C57BL6 (**H**) were produced (**C-H**).

Data are presented as mean ± S.E.M (**A-D**). Sample sizes are as follows: **A-B**: n=17 mice per group; **D-E**: n=3 samples (2 mice per sample), **F-G**: n=2 samples (2 mice per sample), **H**: n=1 sample (2 mice per sample)P-values were determined one-way ANOVA with post hoc Tukey’s test (A, B). P-values are indicated in the figure.

bioRxiv preprint doi: https://doi.org/10.1101/2023.01.26.525731; this version posted September 18, 2024. The copyright holder for this preprint (which was not certified by peer review) is the author/funder, who has granted bioRxiv a license to display the preprint in perpetuity. It is made available under aCC-BY 4.0 International license.

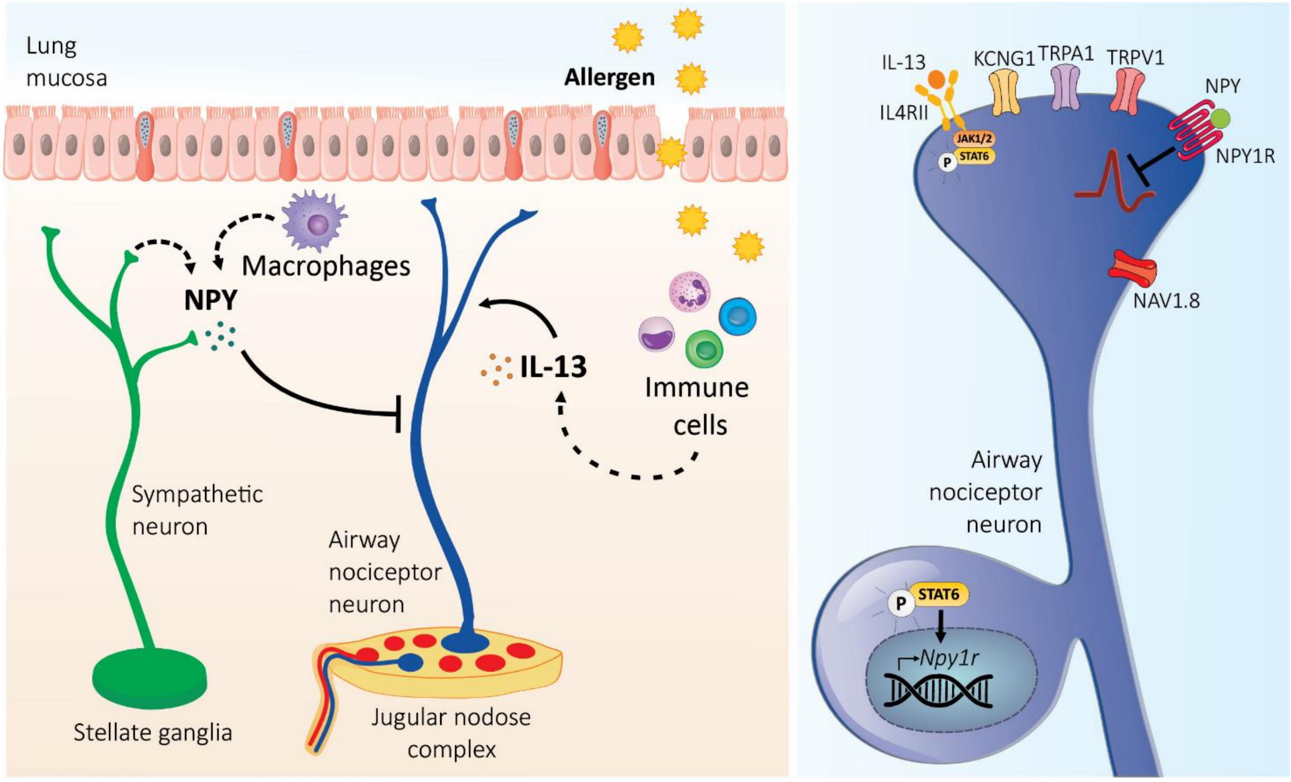

Supplementary figure 9. IL-13 promotes sensory-sympathetic neurons crosstalk in asthma.

When allergens are present in the airways, the immune and stromal cells in the area are activated and release inflammatory cytokines such as IL-13. These cytokines are then detected by nociceptor neurons, which leads to specific changes in gene expression. For example, IL-13 signaling through its interaction with IL4RII leads to increased expression of Npy1r via phosphorylation of STAT6. Via the action of neuropeptide Y (NPY) released by sympathetic neurons and macrophages, this cascade ultimately decreased the sensitivity of NPY1R-expressing nociceptor neurons through reduced intracellular levels of cAMP.

bioRxiv preprint doi: https://doi.org/10.1101/2023.01.26.525731; this version posted September 18, 2024. The copyright holder for this preprint (which was not certified by peer review) is the author/funder, who has granted bioRxiv a license to display the preprint in perpetuity. It is made available under aCC-BY 4.0 International license.

Supplementary Table 1. Airway neuronal subtypes**.** Single cell sequencing data of JNC cells from Prescott et al. were reanalyzed using Seurat. Neuronal cells were selected based on *Slc17a6* (Vglut2) expression. A total of 21 neuronal populations were identified. *Phox2b* and *Prdm12* were used to identify nodose and jugular groups, while nociceptor neurons and low-threshold sensory neurons were defined based on their expression of *Scn10a* and *Scn1a*. The markers identified for each cluster were compared to airway-innervating neurons sequencing by GSEA. A positive normalized enrichment score indicates preferential innervation of the airways for a given neuronal population. Detailed classification of neurons preferentially innervating the airways is displayed in a tab. Other tabs display the average expression of all genes for all neuronal clusters and DESeq2 analysis comparing airway and visceral nociceptor neurons. Another tab shows the average gene expression in clusters identified in reanalysis of projection-seq data from Zhao et al.

Supplementary Table 2. Single cell sequencing of lung immune cells of naïve and AAI mice. 8-week-old female C57BL6 mice underwent the ovalbumin mouse models of asthma. Allergic inflammation was induced in mice by an initial sensitization to ovalbumin (OVA) (i.p. days 0 and 7) followed by inhaled OVA challenges (days 14–17). One day after the last allergen challenge, the lung was harvested, CD45+ cells sorted by flow cytometry before analysis by single cell RNA sequencing. The tables displays the differentially expressed genes induced by AAI, determined using Seurat.

Supplementary Table 3. DESeq2 analysis of JNC cell populations in AAI and Naive conditions. 8-week-old male and female nociceptor neuron reporter (Nav1.8^{cre}::tdTomato$^{fl/wt}$) mice underwent the ovalbumin mouse models of asthma. Allergic inflammation was induced in mice by an initial sensitization to ovalbumin (OVA) (i.p. days 0 and 7) followed by inhaled OVA challenges (days 14–17). On day 2, 3, and 4, mice were injected intranasally with the retrograde tracer DiD' (200 µM). One day after the last allergen challenge, the JNC neurons were harvested for flow cytometry purification and RNA sequencing. DESeq2 pairwise comparison of airway-innervating (Na$_V$1.8$^+$DiD$^+$) naive nociceptor neurons versus AAI nociceptor neurons counterparts shows 92 differentially expressed genes. A literature review for each gene allowed a classification of their expected function [67,108-158]. Other tabs display DESeq2 analysis of visceral nociceptors (Na$_V$1.8$^+$DiD$^+$) and Na$_V$1.8$^-$ cells.

Supplementary Table 4. Cytokines reprograms nociceptor transcriptome. 8-week-old male and female nociceptor neuron reporter (Na$_V$1.8^{cre}::tdTomato$^{fl/wt}$) mice JNC neurons were cultured (24 h) with IL-13 (100 ng/mL), IL-1β (100 ng/mL), BDNF (50 ng/mL), TNF-α (100 ng/mL) or vehicle, before nociceptor purification by flow cytometry and RNA-sequencing. Data from Cobos and colleagues [53] was also reanalyzed by DESeq2 and shows various differentially expressed genes induced in whole DRG three days after spared nerve injury. The different tabs show the DESeq2 analysis for each of these conditions.

Supplementary Table 5. DEGs in IL-13 exposed nociceptors. 8-week-old male and female nociceptor neuron reporter (Na$_V$1.8^{cre}::tdTomato$^{fl/wt}$) mice JNC neurons were cultured (24 h) with IL-13 (100 ng/mL) or vehicle. The nociceptor neurons were then purified by flow cytometry and changes to their transcriptome analyzed by RNA sequencing. The tables display the results of the DESeq2 analysis comparing the vehicle and IL-13-exposed conditions. DEGs are showed in a separate tab. A literature review for each gene allowed a classification of their expected function[67,159-181].

Supplementary Table 6. FPKM normalized sequencing data. Fragment per kilobase per million (FPKM) normalized values for all JNC sequencing data produced in this study (airway nociceptors, visceral nociceptors, Na$_V$1.8$^-$ cells in naive and allergic airway inflammation conditions, cultured nociceptors exposed to vehicle, cytokines, or neurotrophins).

Supplementary Table 7. OVA-exposed Na$_V$1.8^{cre}::NPY1R$^{fl/fl}$ mice show differential gene expression in lung immune cells compared to littermate control. 8-week-old female mice with NPY1R conditional knock-out in nociceptors (Na$_V$1.8^{cre}::NPY1R$^{fl/fl}$) and littermate controls (Na$_V$1.8^{wt}::NPY1R$^{fl/fl}$) underwent the ovalbumin mouse models of asthma. Allergic inflammation was induced in mice (2 mice per samples, 3 samples per group) by an initial sensitization to ovalbumin (OVA) (i.p. days 0 and 7) followed by inhaled OVA challenges (days 14–17). One day after the last allergen challenge, the lung was harvested, CD45+ cells sorted by flow cytometry before analysis by single cell RNA sequencing. To identify robust gene expression changes, a pseudo-bulk approach was used. Cell types were identified using common immune markers. Shown are gene expression averaged for each cell type in each sample identified between OVA-exposed Na$_V$1.8^{wt}::NPY1R$^{fl/fl}$ and Na$_V$1.8^{wt}::NPY1R$^{fl/fl}$ mice.

Supplementary Table 8. OVA-exposed Na$_V$1.8^{cre}::NPY1R$^{fl/fl}$ mice show differential gene expression in lung immune cells compared to littermate control. 8-week-old female mice with NPY1R conditional knock-out in nociceptors (Na$_V$1.8^{cre}::NPY1R$^{fl/fl}$) and littermate controls (Na$_V$1.8^{wt}::NPY1R$^{fl/fl}$) underwent the ovalbumin mouse models of asthma. Allergic inflammation was induced in mice (2 mice per samples, 3 samples per group) by an initial sensitization to ovalbumin (OVA) (i.p. days 0 and 7) followed by inhaled OVA challenges

bioRxiv preprint doi: https://doi.org/10.1101/2023.01.26.525731; this version posted September 18, 2024. The copyright holder for this preprint (which was not certified by peer review) is the author/funder, who has granted bioRxiv a license to display the preprint in perpetuity. It is made available under aCC-BY 4.0 International license.

(days 14–17). One day after the last allergen challenge, the lung was harvested, CD45+ cells sorted by flow cytometry before analysis by single cell RNA sequencing. To identify robust gene expression changes, a pseudo-bulk approach was used. Cell types were identified using common immune markers. Shown are differentially expressed genes identified between OVA-exposed $Na_V1.8^{wt}$::$NPY1R^{fl/fl}$ and $Na_V1.8^{wt}$::$NPY1R^{fl/fl}$ mice.

Supplementary Table 9. OVA-exposed $Na_V1.8^{cre}$::$NPY1R^{fl/fl}$ mice show differential gene set enrichment in lung immune cells compared to littermate control. 8-week-old female mice with NPY1R conditional knock-out in nociceptors ($Na_V1.8^{cre}$::$NPY1R^{fl/fl}$) and littermate controls ($Na_V1.8^{wt}$::$NPY1R^{fl/fl}$) underwent the ovalbumin mouse models of asthma. Allergic inflammation was induced in mice (2 mice per samples, 3 samples per group) by an initial sensitization to ovalbumin (OVA) (i.p. days 0 and 7) followed by inhaled OVA challenges (days 14–17). One day after the last allergen challenge, the lung was harvested, CD45+ cells sorted by flow cytometry before analysis by single cell RNA sequencing. To identify robust gene expression changes, a pseudo-bulk approach was used. Cell types were identified using common immune markers. Shown are GO gene set enrichment for each cell type identified between OVA-exposed $Na_V1.8^{wt}$::$NPY1R^{fl/fl}$ and $Na_V1.8^{wt}$::$NPY1R^{fl/fl}$ mice.

Supplementary Table 10. T cell subpopulations analysis. T cells were subsetted from the CD45+ single cell RNA sequencing data. High resolution clustering revealed several clusters of T cells and innate lymphoid cells, including CD4, CD8, iT-cells (immature T cells), ILCs, NKT, and γδT cells. The table displays average expression in each of those lymphocytes cell subtypes, in each sample, as well as the top markers of each cluster.
